# Nomogram for estimating the risk of suicide attempts in major depressive disorder: integrating demographic, clinical and biochemical markers – a cross-sectional study

**DOI:** 10.3389/fpsyt.2025.1634164

**Published:** 2025-09-19

**Authors:** Nan Lyu, Qian Zhao, Rina Dutta, Han Wang, Gang Wang, Allan H. Young

**Affiliations:** ^1^ Beijing Key Laboratory of Mental Disorders, National Clinical Research Center for Mental Disorders & National Center for Mental Disorders, Beijing Anding Hospital, Capital Medical University, Beijing, China; ^2^ Advanced Innovation Center for Human Brain Protection, Capital Medical University, Beijing, China; ^3^ Laboratory for Clinical Medicine, Capital Medical University, Beijing, China; ^4^ Department of Psychological Medicine, Institute of Psychiatry, Psychology and Neuroscience, King’s College London, London, United Kingdom; ^5^ South London and Maudsley NHS Foundation Trust, Bethlem Royal Hospital, Beckenham, Kent, United Kingdom

**Keywords:** major depressive disorder, suicide attempts, biomarkers, folate, nomogram

## Abstract

**Background:**

Major depressive disorder (MDD) is a significant risk factor for suicide attempts (SA), making early identification of those most at risk crucial for effective clinical intervention. This study aimed to identify demographic, clinical, and biochemical correlates of SA among inpatients with MDD.

**Methods:**

In this cross-sectional study, data were collected from 968 inpatients diagnosed with MDD, including 392 with documented suicide attempts (MDD-SA) and 576 without suicidal behaviors (MDD-NSA). Demographic characteristics, clinical history, and peripheral biochemical markers were analyzed using multivariable logistic regression to assess associations with SA. Variable selection was performed using penalized logistic regression with 10-fold cross-validation, and the selected variables were then entered into a binary logistic regression model to illustrate the relative contribution of significant factors. Model performance was evaluated using the area under the Receiver Operating Characteristic (ROC) curve, calibration plots, and decision curve analysis (DCA).

**Results:**

Significant differences were found between MDD-S and MDD-N in marital status (42.3% vs. 57.7%, p = 0.004), alcohol consumption (15.82% vs. 8.85%, p < 0.001), tobacco use (20.67% vs. 13.73%, p = 0.004), education level (p < 0.001), life events (79.59% vs. 65.28%, p < 0.001), and folate levels (p < 0.001). MDD-S patients were more likely to undergo modified electroconvulsive therapy (61.73% vs. 39.41%, p < 0.001) and mood stabilizers (26.02% vs. 18.92%, p = 0.009). The nomogram incorporated tobacco use, alcohol consumption, education level, life events, and folate levels, showing good discrimination (C-index = 0.709, bootstrap = 0.703). ROC analysis demonstrated an AUC of 0.709 (95% CI: 0.677–0.742), and DCA confirmed clinical utility.

**Conclusion:**

Several routinely available variables, including tobacco use, alcohol consumption, education level, life events, and folate level, were associated with suicide attempts in MDD inpatients. Our findings highlight these important correlates, which may help clinicians in recognizing patients at elevated risk. The nomogram provided in this study should be regarded only as a visualization to better illustrate the contribution of these factors, rather than as a clinical assessment tool. Prospective multicenter studies are needed for further validation.

## 1 Introduction

Major depressive disorder (MDD) is a common mental health condition associated with significant morbidity and mortality including suicide attempts (SA) which are a leading cause of death in individuals with depression (1). According to the World Health Organization, suicide accounts for nearly 800,000 deaths annually, and it remains one of the top causes of death globally (2). MDD is recognized as a major contributing factor to suicidal behaviors and the risk is heightened when patients experience recurrent depressive episodes, substance abuse, or comorbid psychiatric conditions (3). Accurate prediction and timely intervention are crucial for reducing the risk of suicide in MDD patients, but prevention efforts remain limited by the difficulty of identifying high-risk individuals.

Guided by the biopsychosocial model (4) and the stress–diathesis model (5) of suicidal behavior, which posit that biological vulnerability interacts with clinical symptoms and psychosocial stressors to precipitate suicidal acts, within this framework, risk factors from different domains can be understood as interacting components of a unified risk profile. For instance, lower education levels, lack of social support, and substance use have been linked to an increased risk of SA (6). These demographic factors interact with psychological vulnerability and social stressors, exacerbating the risk of suicidal behaviors (6). In parallel, certain clinical features play a pivotal role in predicting suicidal behaviors. Features such as the severity of depressive symptoms, the number of depressive episodes, and the presence of psychotic symptoms have been strongly associated with an increased risk of SA (7). Age of onset and duration of depression can also provide insight into the chronicity of the disorder, which is a key element influencing suicidal tendencies (8, 9).

At the biological level, mechanisms such as hypothalamic–pituitary–adrenal (HPA) axis dysregulation and impaired one-carbon metabolism may shape vulnerability by altering mood regulation and stress reactivity (10, 11). From an integrated perspective, these demographic, clinical, and biological domains should not be viewed in isolation but rather as interacting components of a unified suicide risk profile (7, 12). For example, stressful life events can activate the HPA axis, leading to abnormal adrenocorticotropic hormone (ACTH) secretion, which has been observed in individuals at high suicide risk (13), This dysregulation, combined with exposure to significant life stressors, may be a potent driver of suicidal behavior.

Deficiencies in folate—a key cofactor in neurotransmitter synthesis—can impair neural resilience (14).Moreover, both alcohol consumption and tobacco use have been shown to lower folate levels and disrupt its metabolism, potentially compounding the neurobiological effects of folate deficiency (15–17). In addition to their impact on folate status, alcohol and tobacco use may intensify both biological stress responses and psychosocial disadvantage (18, 19). Elevated homocysteine levels have been linked to neurotoxicity and increased oxidative stress, which may contribute to the onset of depression and suicidal tendencies (20). On the other hand, folate, which plays a crucial role in homocysteine metabolism, has been found to be deficient in individuals with severe depression and suicidal behaviors (21). Folate supplementation has shown promise in reducing homocysteine levels, which could mitigate the risk of SA by addressing underlying neurobiological mechanisms (22).

Given the complexity of suicide risk, nomograms have emerged as valuable tools for integrating multiple variables, both clinical and biological, into a single model. Nomograms offer a visual representation that allows clinicians to make individualized risk assessments based on patient-specific data (23–25), However, few studies have combined biological indicators with clinical treatment factors to explore their joint role in suicide risk prediction.

Based on previous evidence, we hypothesized that: (1) specific demographic characteristics (e.g., lower education, alcohol and tobacco use, recent life events), clinical features, and biochemical markers (folate, ACTH and homocysteine levels) would each be independently associated with SA in MDD; and (2) integrating variables from the domains into a single nomogram would provide a more comprehensive tool for estimating the probability of SA.

Building on previous work from our institution that developed a nomogram-based model using only demographic and clinical variables (25), the present study extends this approach by incorporating biochemical markers and analyzing a larger inpatient cohort. The nomogram in this study serves as a simple visual tool to integrate these factors, emphasizing their importance for comprehensive clinical evaluation rather than providing a precise numeric risk estimate. Therefore, the aim of this study was to identify key demographic, clinical, and biochemical correlates of SA in MDD inpatients. In addition, we constructed an exploratory nomogram to provide a visual illustration of the relative contribution of these factors, which may offer a framework for future research but is not intended for direct clinical application.

## 2 Materials and methods

### 2.1 Participants

This was a retrospective cross-sectional study conducted at Beijing Anding Hospital, Capital Medical University, between January 2013 and December 2020. Participants were diagnosed with MDD following the International Classification of Diseases, Tenth Revision (ICD-10) (26) guidelines by two experienced psychiatrists. All patients included in the study had complete medical records and were followed for one-year post-discharge to monitor recurrence and rehospitalization rates. Exclusion criteria were: (1) those diagnosed with other mental disorders, such as schizophrenia and bipolar disorder; (2) severe physical diseases including severe hepatic injury (e.g., hepatic failure or decompensated cirrhosis), advanced renal impairment, heart failure, and other severe systemic conditions; (3) long-term hospitalized patients (>6 months); (4) patients with drug or alcohol dependence or abuse issues; (5) women who were pregnant and/or lactating; and (6) incomplete medical records, particularly the absence of detailed descriptions and assessments related to SA. (6) unclear or insufficient documentation regarding the presence or absence of SA in the medical records, making group classification unreliable.

Patients were divided into two groups based on the presence of suicidal behaviors within three months prior to admission, as indicated in their medical records. Those with documented SA were placed in the MDD with SA group (MDD-S), while those without SA formed the non-suicidal group (MDD-N). For patients in the MDD-S group, the specific method of SA was extracted from the “present illness” section of the medical records, with the most recent attempt prior to admission being coded. As this was a retrospective study, SA was clearly documented in the medical records. However, documentation of suicidal ideation varied widely in detail and clarity, making it difficult to reliably determine whether patients had clear suicidal thoughts at the time. Furthermore, SA, as a severe phenotype, may be more useful for identifying potential biological markers and has greater clinical warning significance. Therefore, patients with suicidal ideation were not included in this study.

### 2.2 Clinical variables

Baseline variables were extracted from patients’ medical records at the time of the index hospitalization. These included demographic characteristics (age, sex, marital status, education level, occupation), clinical features (tobacco use, alcohol consumption, recent life events, recurrence status, presence of psychotic symptoms, comorbid chronic physical diseases), treatment history (antidepressants, mood stabilizers, antipsychotics, and electroconvulsive therapy), and biochemical indicators (folate, ACTH, and homocysteine).Depression severity was classified according to the ICD-10 diagnostic system, which distinguishes mild, moderate, and severe depression. Since mild cases were not admitted as inpatients, only moderate and severe depression were present in this cohort. Therefore, depression severity was categorized into two levels (moderate vs. severe) based on the clinical diagnosis. SA method was extracted from the “present illness” section of medical records, with the primary method used in the most recent attempt before admission being coded. Abnormal liver function, extracted from admission diagnoses, was included as a binary variable to control for potential confounding effects on biochemical markers, particularly folate and homocysteine levels. Follow-up information during the one-year period after discharge, including emergency department visits and readmissions, was also collected retrospectively. These follow-up data were not included in the binary logistic model but were analyzed descriptively to observe post-discharge rehospitalization rates.

### 2.3 Ethics approval and consent to participate

The study was approved by the Ethics Committee of Beijing Anding Hospital (approval number: 2022–133) and conducted in accordance with the Declaration of Helsinki. As this was a retrospective study using anonymized clinical data, the requirement for written informed consent was waived. Verbal informed consent was obtained by phone from patients when possible, and others had signed a broad consent form at admission permitting the use of their clinical data for research. No identifiable personal information was included in the analysis.

### 2.4 Statistical analysis

Data were processed using R software (version 4.4.1) and SPSS (version 27.0). Categorical variables were transformed into factors, while continuous variables were converted into numeric values to facilitate analysis. Summary statistics were used to describe the demographic, clinical, and biochemical characteristics of the two groups. Differences between the MDD-S and MDD-N groups were assessed using the Chi-Square Test for categorical variables and the Wilcoxon Rank-Sum Test for non-normally distributed numerical variables.

Univariate and multivariable logistic regression analyses were performed to identify associated factors of SA. Independent variables included demographic details, clinical characteristics, and blood biomarkers. In data analysis, tobacco use is categorized as 1 for frequent smokers and 0 for non-smokers or occasional smokers. Similarly, alcohol consumption is represented as 1 for habitual drinkers and 0 for those who do not drink or drink occasionally. Education is classified as 0 for middle school or below (≤9 years, 1 for high school (9~12 years, and 2 for university-level education and above (≥12 years. Life events, referring to significant experiences prior to a depressive episode, are coded as 1 for “yes” and 0 for “no”. The folate level is directly entered as its measured value in ng/mL. The”abnormal liver function” was extracted from admission diagnoses and was defined as one or more liver-related laboratory values exceeding the upper normal limit (ALT or AST > 40 U/L, ALP > 125 U/L, GGT > 60 U/L, total bilirubin > 20.5 μmol/L, or albumin < 35 g/L, with albumin and INR used to assess hepatic synthetic function). This variable was coded as a binary variable and included in both univariable and multivariable logistic regression analyses.

The results were expressed as odds ratios (OR) with 95% confidence intervals (CI), and p-values below 0.05 were considered statistically significant. Variable selection for the final model was based on a backward stepwise method. Two-tailed P values were used for all statistical analyses, and p < 0.05 was considered as statistically significant.

### 2.5 Model construction and evaluation

To identify the best combination of model parameters (alpha and lambda), a 10-fold cross-validation was performed using the caret package in R (27). A parameter grid was defined, with alpha ranging from 0 to 1 in increments of 0.1, and lambda ranging from 0 to 1 in increments of 0.1. The train function was used to perform the cross-validation and select the optimal parameters based on the highest accuracy. Using the optimal parameters (alpha = 0.3 and lambda = 0.1) identified from the cross-validation, a final binary logistic regression model fitted using the lrm function in the rms package in R (28). This final model was used to construct the nomogram. A nomogram is a graphical tool that transforms a regression model into a visual scoring system, allowing the probability of an event to be estimated for an individual by summing the scores for each variable, and providing a more intuitive and clinically applicable format for individualized risk assessment compared with presenting only regression coefficients or odds ratios.

An exploratory nomogram was then generated using the key variables identified in the 10-fold cross-validation process, with each variable assigned a corresponding score on a scale of 0 to 100. These scores were combined to generate an overall score for each patient. The total score was then transformed into a logit, which was used to calculate the individual probability of SA ranging from 0% to 100%. The model’s discriminative performance was assessed using Harrell’s concordance index (C-index) and calibration plots (28, 29). Generally, C-index greater than 0.7 indicates a well-calibrated model. To internally validate the model, the bootstrap method was employed. The Hosmer–Lemeshow test (30) was applied to evaluate the goodness of fit, with a p-value greater than 0.05 suggesting a good fit. Additionally, variance inflation factors (VIF) (31) were used to check for multicollinearity among the variables in the model, where a VIF above 10 suggests significant multicollinearity.

To evaluate the accuracy of the nomogram, a calibration curve was plotted, which compares the predicted probabilities from the model to the actual observed outcomes. This curve reflects how closely the model’s estimated risks align with reality. The model’s ability to differentiate between patients with and without SA was further assessed using a Receiver Operating Characteristic (ROC) curve. The ROC curve plots sensitivity (true positive rate) against 1 - specificity (false positive rate) across various threshold levels, and the Area Under the Curve (AUC) was calculated to provide a single metric of the model’s discriminative power. Additionally, the clinical utility of the nomogram was determined through decision curve analysis (DCA) (32). DCA estimates the net benefit of the model by weighing the true positives against the false positives, and compares these results to hypothetical “treat all” or “treat none” strategies across a range of threshold probabilities.

## 3 Results

### 3.1 Baseline demographic, clinical characteristics, and biochemical parameters

A detailed comparison of demographic, clinical characteristics, and peripheral blood indicators between the MDD-S and MDD-N groups is shown in **Table 1**. Notably, the proportion of married individuals in the MDD-S group is significantly lower (42.3% vs. 57.7%, p = 0.004). Additionally, alcohol and tobacco use are higher in the MDD-S group, with significant differences in alcohol consumption (p < 0.001) and tobacco use (p = 0.004). Furthermore, a significantly higher percentage of individuals in the MDD-S group experienced life events (79.6% vs. 65.3%, p < 0.001). Patients in the MDD-S group also had a higher proportion of severe depression compared to the MDD-N group (92.1% vs. 89.6%, p = 0.048).There were no significant differences between the two groups in terms of age (p = 0.071), gender (p = 0.471), occupation (p=0.077), duration of illness (p = 0.802), proportion of recurrence (p = 0.874), nor the presence of psychosis (p = 0.300). The distribution of SA methods among patients in the MDD-S group is provided.

**Table 1:** Demographic, clinical, and biochemical characteristics of MDD patients with SA and without SA.

Variables	MDD-S (n = 392)	MDD-N (n = 576)	Z/χ2	*P* value
Demographic characteristic				
Age (years)	29 (18, 53)	34 (19, 53)	-1.80	0.07
Female (%)	288 (73.47)	411 (71.35)	0.52	0.47
Marital state (yes, %)	166 (42.35)	266 (57.73)	8.24	**0.004**
Alcohol consumption (yes, %)	62 (15.82)	51 (8.85)	10.97	**<0.001**
Tobacco use (yes, %)	81 (20.67)	79 (13.73)	8.16	**0.004**
Occupation			5.13	0.08
Student (yes, %)	122 (31.12)	146 (25.35)		
Employed (yes, %)	237 (60.46)	389 (67.53)		
Unemployed (yes, %)	33 (8.42)	41 (7.12)		
Level of education (n, %)			85.27	**<0.001**
middle school and below	85 (21.68)	224 (38.89)		
high school	219 (55.87)	153 (26.56)		
University and above	88 (22.45)	199 (34.55)		
Clinical characteristics				
Duration (years)	2.00 (0.67, 7.00)	2.00 (0.58, 7.00)	-0.25	0.80
Age of onset (years)	23 (15, 41)	28 (16, 45)	-1.94	0.05
Life events (yes, %)	312 (79.59)	376 (65.28)	23.25	**<0.001**
Recurrence (yes, %)	179 (45.66)	266 (46.22)	0.03	0.87
Psychosis (yes, %)	107 (27.30)	175 (30.38)	1.08	0.30
Depression severity (n, %)			3.89	**0.048**
Moderate depression	26 (6.63)	60 (10.42)		
Severe depression	361 (92.10)	516 (89.58)		
Chronic physical diseases (yes, %)	30 (7.65)	44 (7.64)	0.52	0.47
Abnormal liver function (yes, %)	13 (3.32)	21 (3.65)	0.08	0.79
Treatment and outcome				
MECT (yes, %)	242 (61.73)	227 (39.41)	46.55	**<0.001**
Mood stabilizer (yes, %)	102 (26.02)	109 (18.92)	6.89	**0.009**
Antipsychotics (yes, %)	254 (64.80)	386 (67.01)	0.51	0.47
Readmission rate within 180 days	48 (12.24)	68 (11.81)	0.04	0.84
Readmission rate within 1 year	29 (7.40)	30 (5.21)	1.95	0.16
Peripheral blood indicators				
Folate (ng/mL)	4.98 (3.19, 7.06)	5.62 (3.97, 8.76)	-4.01	**<0.001**
ACTH (pg/mL)	40.55 (26.93, 59.50)	41.95 (27.00, 63.10)	-0.73	0.46
Homocysteine (μmol/L)	13.28 (11.20, 16.95)	13.20 (11.21, 16.80)	0.48	0.63

Continuous variables are presented as median (lower quartile, upper quartile) and compared using the Kruskal–Wallis H test. Categorical variables are presented as n (%) and compared using the Chi-square test. MDD, major depressive disorder; SA, suicide attempt; ACTH, adrenocorticotropic hormone; MECT, modified electroconvulsive therapy.

Bold values indicate statistical significance (p < 0.05).

Clinically, patients in the MDD-S group were more likely to receive modified electroconvulsive therapy (61.7% vs. 39.4%, p < 0.001) and mood stabilizers (26.0% vs. 18.9%, p = 0.009). However, there were no significant differences in the use of antipsychotics between the groups. Biochemically, folate levels were significantly lower in the MDD-S group (p < 0.001), while no notable differences were observed for ACTH or homocysteine levels between the 2 groups.

### 3.2 Identification of associated factors

**Figure 1:**
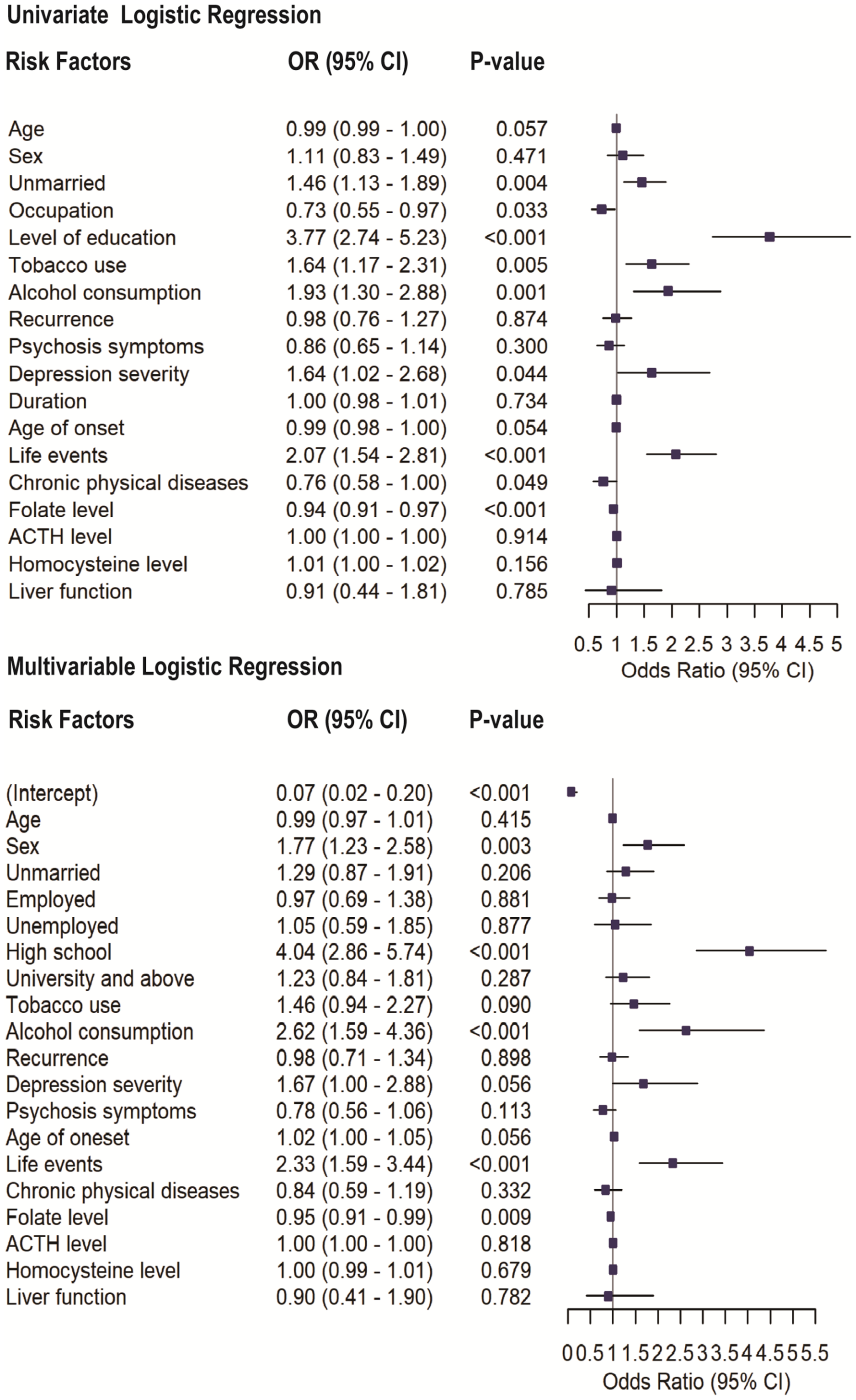
Univariate and multivariable logistic regression analyses assessing the association between selected variables and SA in patients with MDD. Odds ratios (ORs) with 95% confidence intervals (CIs) and corresponding p-values are shown for each variable.

The results of the univariate and multivariable logistic regression analyses presented in **Figure 1** highlight variables associated with SA in patients with MDD. In the univariate analysis, significant variables of SA include being unmarried (OR = 1.46, p = 0.004), tobacco use (OR = 1.64, p = 0.005), alcohol consumption (OR = 1.93, p = 0.001), life events (OR = 2.07, p<0.001), occupation (OR = 0.73, p=0.03), and levels of education (OR = 3.77, p<0.001). Depression severity (OR = 1.68, 95% CI: 1.01–2.79, p = 0.048), chronic physical diseases (OR = 0.76, 95% CI: 0.58–1.00, p = 0.049), and Folate levels (OR = 0.94, p < 0.001) were also significantly associated with SA. However, other variables, such as recurrence, liver function and the presence of psychotic symptoms, did not exhibit significant associations with SA (p > 0.05).

In the multivariable logistic regression analysis, all variables included in the univariate analysis were entered as covariates. After adjustment, high school educational levels (OR = 4.10, p < 0.001), alcohol consumption (OR = 2.57, p < 0.001), and experiencing life events (OR = 2.32, p < 0.001) remained significantly associated with SA. Additionally, sex (OR = 1.74, p = 0.003) and lower folate levels (OR = 0.95, p = 0.012) also emerged as significant contributors. Depression severity showed only a marginal association with SA (OR = 1.67, 95% CI: 1.00–2.88, p = 0.056) and was not retained as an independent factor. However, factors such as being unmarried and tobacco use, which were statistically significant in the univariate analysis, lost their significance in the multivariable model.

Using a 10-fold cross-validation with the caret package in R, the optimal model parameters (alpha = 0.3 and lambda = 0.1) were identified from a predefined grid. Based on these parameters, a logistic regression model was fitted using the glmnet package in R (33). The following variables were selected with their corresponding regression coefficients β: Tobacco use (β = 0.033), alcohol consumption (β = 0.241), folate level (β = −0.015), life events (β = 0.301), and education level (β = 0.749).

### 3.3 Exploratory nomogram

**Figure 2:**
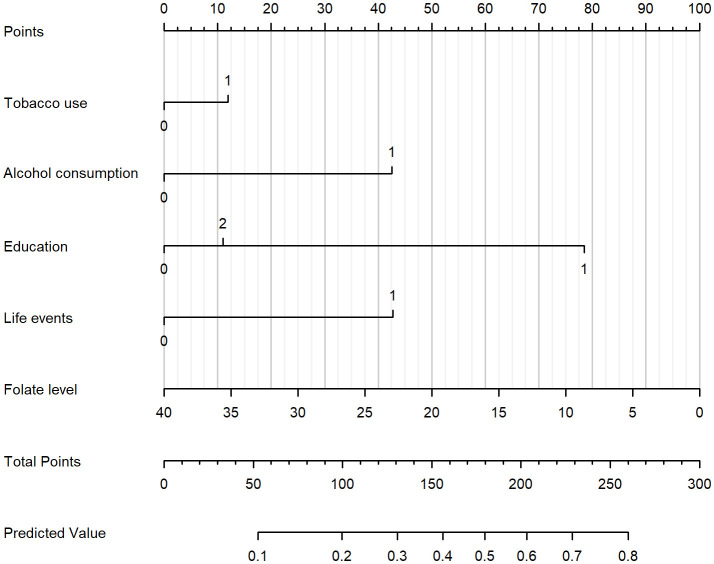
Nomogram for estimating the probability of SA in patients with MDD. The nomogram incorporates tobacco use, alcohol consumption, education level, life events, and folate level. To use the nomogram, draw a vertical line from each variable to the “Points” scale to determine the score for that variable. Sum all scores to obtain the “Total Points,” and then project downward to the “Predicted Value” scale to estimate the individual probability of SA.

**Figure 3:**
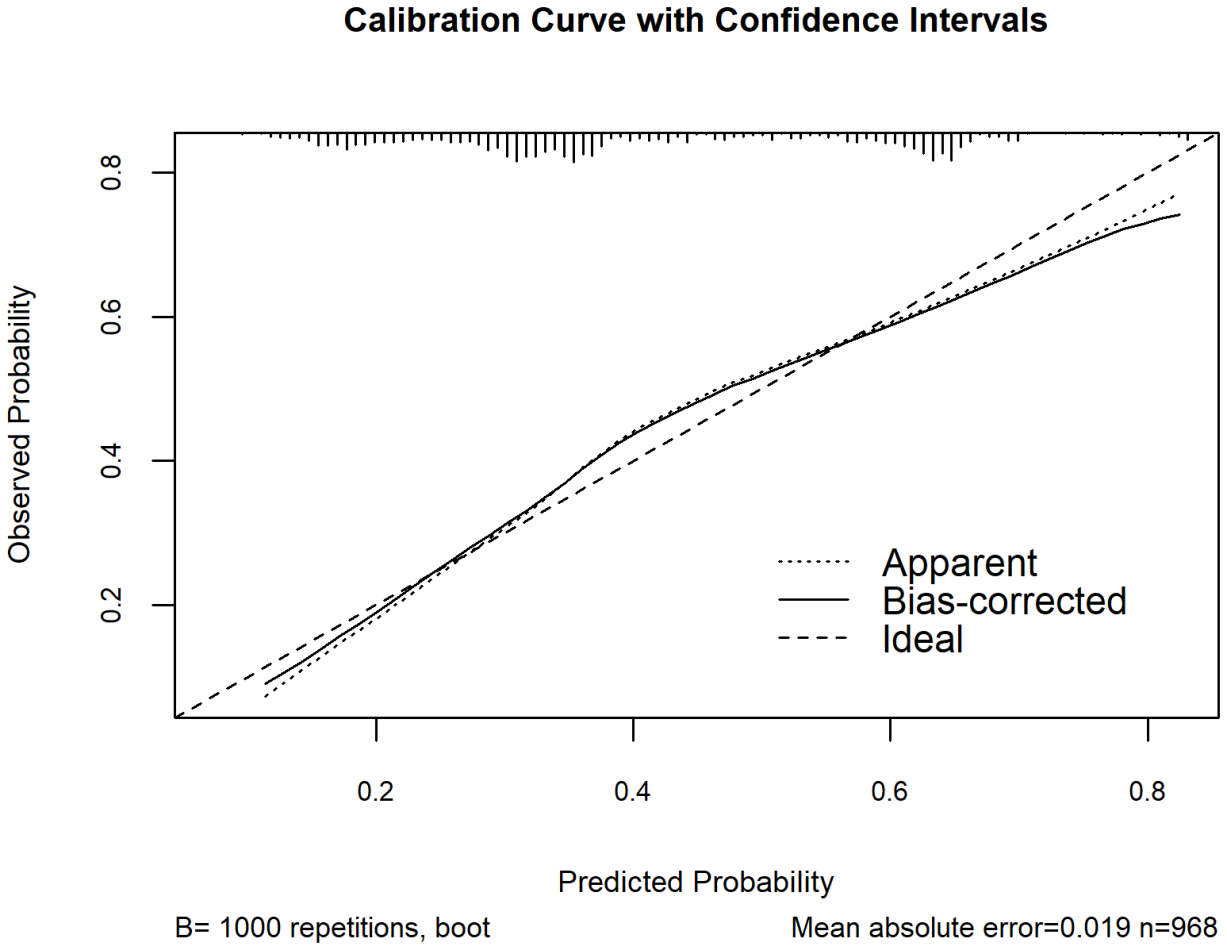
Calibration curve of the nomogram for assessing the agreement between model-estimated and observed probabilities of SA among MDD patients. The x-axis represents the model-estimated probability of SA from the nomogram, and the y-axis represents the observed probability after bootstrap resampling (B = 1000). The dotted line (“Apparent”) shows the model’s performance on the original dataset, the solid line (“Bias-corrected”) shows the performance after bootstrap correction, and the dashed line (“Ideal”) represents perfect calibration.

Based on **Figure 2**, a nomogram was constructed to visualize the relative contribution of significant factors associated with SA in patients with MDD. The model was developed using binary logistic regression and incorporated variables selected from the multivariable logistic regression analysis and 10-fold cross-validation, such as tobacco use, alcohol consumption, education level, life events, and folate levels. For instance, a patient who frequently smokes (12.5 points), habitually drinks alcohol (42.5 points), has a high school education (79 points), experienced life events before the onset of depression (42.5 points) and has a folate level of 5 ng/mL (87.5 points) would have a total score of 265. This score corresponds to an estimated 80% probability of having had an SA. The total score from the nomogram reflects the individual’s assessed probability of SA. The calibration curve (**Figure 3**) showed that the predicted probabilities align well with the observed outcomes, indicating a high level of accuracy in the model’s discrimination. The C-index of the nomogram was 0.709, demonstrating good discriminative ability, and it slightly decreased to 0.703 after internal validation using the bootstrap method, confirming the robustness of the model.

**Figure 4:**
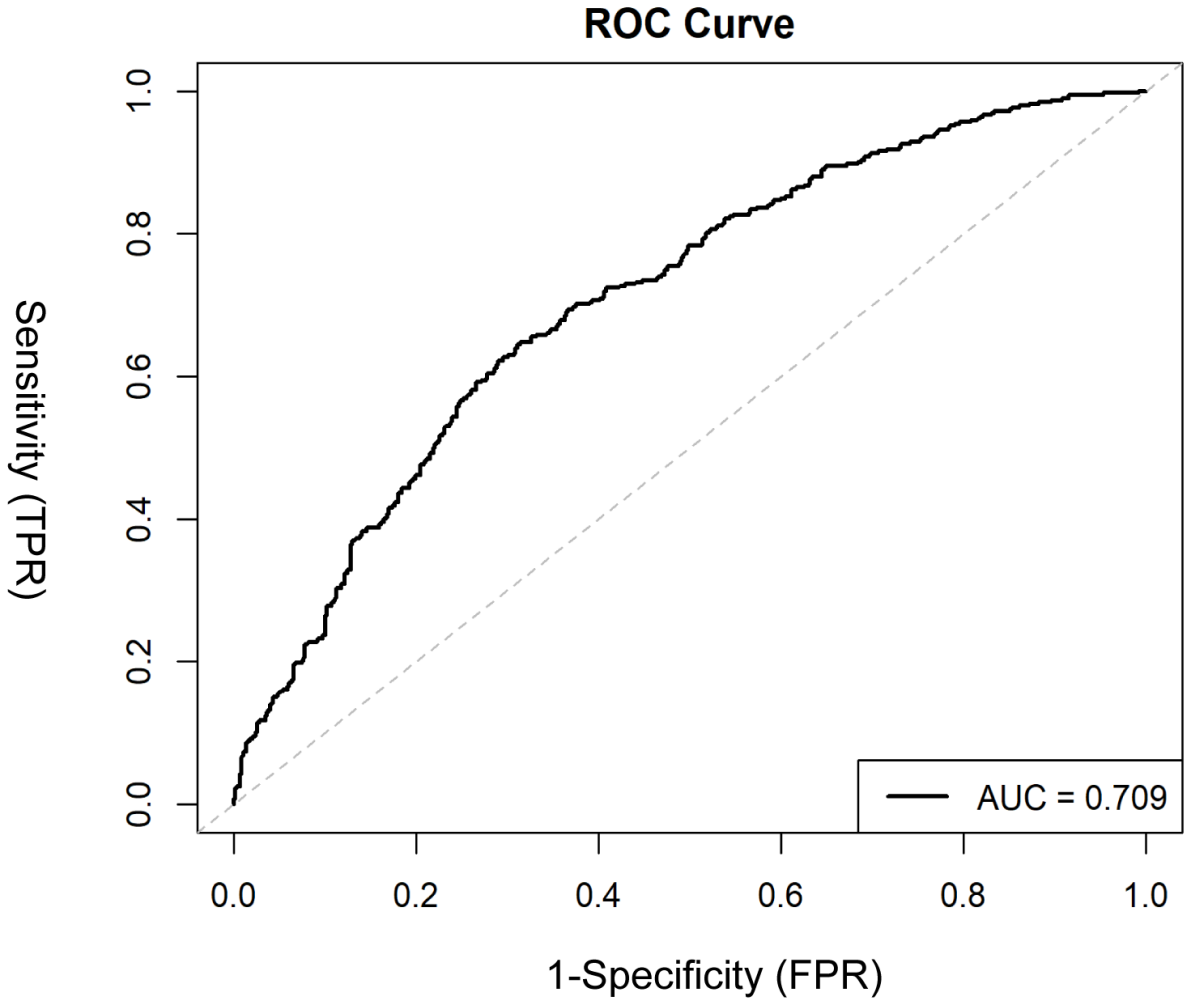
ROC curve evaluating the discriminative ability of the nomogram in distinguishing MDD patients with and without SA. The x-axis represents 1 – specificity (false positive rate), and the y-axis represents sensitivity (true positive rate). The area under the curve (AUC) is shown, with a higher AUC indicating better discrimination.

**Figure 5:**
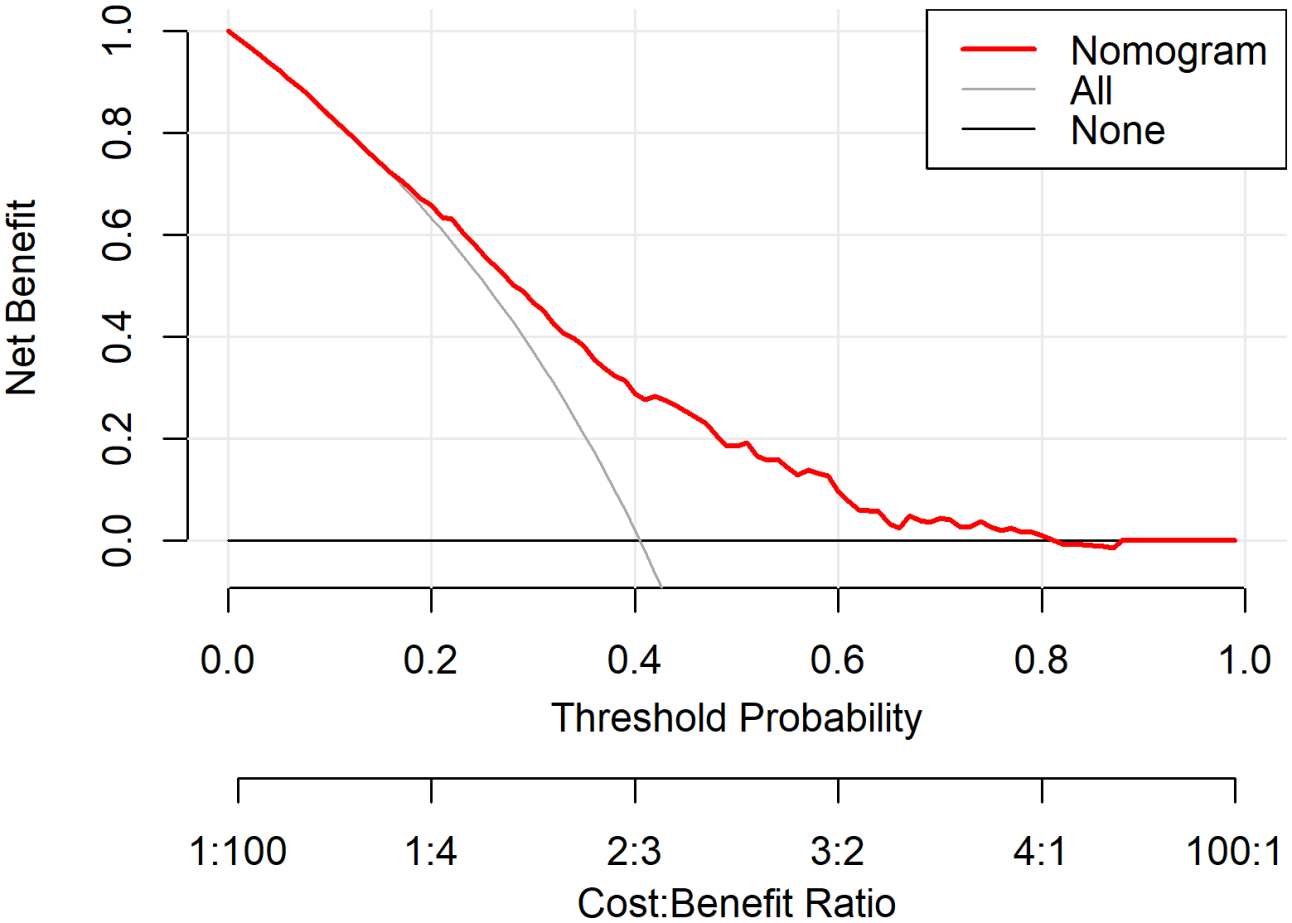
DCA evaluating the net benefit of the nomogram in differentiating MDD patients with and without SA. The x-axis represents the threshold probability at which a clinician might decide to take action, and the y-axis represents the corresponding net benefit. The red line (“Nomogram”) indicates the model-based assessment, the grey line (“All”) assumes all patients have SA, and the black line (“None”) assumes no patients have SA.

The Model discrimination was further supported by the ROC curve, with an AUC of 0.709 (95% CI: 0.677–0.742, p < 0.001) (**Figure 4**). Additionally, the Hosmer–Lemeshow test result (p = 0.30) suggests a good fit for the model. Multicollinearity among the variables was assessed using variance inflation factors (VIF), and all values were below 10, indicating issues with multicollinearity in the model were unlikely. The decision curve analysis (**Figure 5**) shows that the nomogram provides a clear net benefit compared to the “treat all” or “treat none” strategies, particularly for threshold probabilities between 0.1 and 0.6.

## 4 Discussion

This study identified several key factors associated with SA in patients with MDD. The SA group exhibited a higher proportion of unmarried and unemployed individuals, higher rates of tobacco and alcohol use, and lower educational levels. Significant life events were more common among these patients, suggesting a strong link to suicide risk. Biochemically, folate levels were notably lower in the SA group, indicating a potential metabolic component to suicidal behaviors. Clinically, patients with SA were more likely to receive modified electroconvulsive therapy (MECT) and mood stabilizers, likely reflecting more severe depressive episodes. To illustrate the combined impact of these factors, we constructed an exploratory nomogram, which provides a visual representation of their relative contributions. Our findings therefore highlight a set of routinely available demographic, clinical, and biochemical variables that warrant particular attention in the assessment of suicide risk among MDD inpatients.

From a clinical perspective, these findings may serve as indicators to alert clinicians to patients who require closer monitoring, enhanced psychosocial support, and targeted safety planning during hospitalization (34, 35). For example, screening for recent life events and substance use should be a standard component of admission evaluations, as these factors may indicate heightened vulnerability to suicidal behavior. Similarly, low folate levels, which can be easily obtained through standard laboratory tests, might prompt more comprehensive nutritional assessments and interventions. The nomogram in this study offers a simple visual tool to combine these factors together. The nomogram in this study serves as a simple visual tool to integrate these factors, emphasizing their importance for comprehensive clinical evaluation rather than providing a precise numeric risk estimate.

Our study shares similarities with prior research in identifying both clinical and demographic factors for SA in patients with MDD. For instance, several studies have found that demographic factors such as being unmarried, and lower educational levels are associated with an increased risk of SA in MDD patients, consistent with our findings. These results align not only with studies from Asian populations (6, 11, 36, 37), but also with evidence from Western cohorts. Large register-based studies in Norway (38) and studies from Europe and America (39, 40) have reported similar associations, suggesting that marital status and education level may represent cross-cultural risk factors for suicidality.

Interestingly, our data suggest that individuals with a high school education may represent a particularly vulnerable group in the Chinese context, whereas those with either middle school or lower education and those with a university-level or higher education did not show a similarly elevated risk. One possible explanation could be that individuals with a moderate level of education often struggle to adjust their economic and social expectations, placing them in a challenging situation. They may lack the emotional coping strategies that those with higher education possess, and the gap between their expectations and reality can increase psychological pressure (9, 41). This dual dilemma may elevate their risk of suicide, warranting greater attention in clinical interventions.

Smoking and alcohol consumption were also identified as significant associated factors for SA in our study. A strong association between substance use and increased suicide risk have been reported by several studies (18, 19). Smoking, often linked with higher levels of psychological distress and substance dependence, can exacerbate depressive symptoms and contribute to impulsive behaviors, thereby increasing the risk of SA (42). Additionally, alcohol consumption is known to impair judgment, disinhibit behaviors and exacerbate hopelessness and impulsivity, which are key components of suicidal behaviors (43). Furthermore, individuals who smoke or consume alcohol regularly may experience greater social isolation, leading to a lack of support systems that could otherwise mitigate suicidal tendencies (44). These findings underscore the importance of incorporating substance use assessments into routine clinical evaluations for inpatients with MDD, consistent with evidence from both East Asian (19, 42) and Western populations (45, 46) that substance use is associated with suicidal behavior.

Our study also underscores the critical role of stressful life events in relation to suicidal behaviors. This finding aligns with previous research demonstrating that stressful events significantly increase the risk of subsequent suicidal behaviors in Asian and Western populations (12, 47), even in the short term (48). Stressful life events—such as the loss of a loved one, financial difficulties, and interpersonal conflicts—are well-documented to exacerbate depressive symptoms and elevate the likelihood of SA, a cross-culturally relevant contributor to suicidal behavior, highlighting their cross-cultural relevance in understanding suicidal behavior. These adverse experiences can induce chronic stress, activating neurobiological mechanisms such as the hypothalamic-pituitary-adrenal (HPA) axis, which may increase vulnerability to suicidal thoughts and attempts (49). Chronic activation of the HPA axis has been shown to raise levels of adrenocorticotropic hormone (ACTH), which in turn influences cortisol release and can exacerbate the physiological and psychological stress response (50, 51).

In our study, we did not find a significant association between peripheral blood ACTH levels and SA. The existing literature on this topic is similarly inconsistent: while some studies have reported a relationship between ACTH and suicidal behaviors (13, 52, 53), others have found no significant association (54, 55). This inconsistency reflects the complex interplay between ACTH levels and suicidal behaviors, which may be influenced by the heterogeneity of suicidal behaviors themselves. Additionally, the dynamic fluctuations of ACTH, variability in HPA axis sensitivity, and differences in population characteristics could contribute to the observed lack of correlation. It is also plausible that other downstream factors, such as cortisol levels or the sensitivity of HPA axis feedback mechanisms, may play a more direct role in modulating the risk of SA.

In terms of clinical characteristics, our study found no significant differences in the age of onset of MDD. Previous research has shown that early-onset depression is strongly associated with a more severe illness trajectory, including higher rates of recurrence, comorbid psychiatric conditions, and increased suicide risk (8, 9). While our findings did not reveal a clear distinction, the borderline p-value (p=0.052) suggests that the age of onset may still have a predictive influence on SA. This subtle statistical trend underscores the need for further research to better understand its potential role.

Similarly, regarding psychotic symptoms, we did not find a significant association with SA. Previous research has provided mixed evidence: some studies have linked psychotic symptoms, such as hallucinations and delusions, to increased suicide risk (56, 57), while others have not supported this association (58, 59). The lack of association in our cohort may also reflect the characteristics of the inpatient population, who typically present with more severe depression, potentially overshadowing the predictive value of psychotic symptoms. These findings suggest that psychotic symptoms may have limited utility as a variable for distinguishing patients with and without SA in this context.

With respect to depression severity, we observed a significant association with SA in the univariate analysis; however, this effect weakened to a marginal level in the multivariable model. This attenuation likely reflects the inpatient sample characteristics, as the majority of patients were admitted with severe depression, leading to a skewed distribution and reduced statistical power for this variable. Nevertheless, evidence from broader clinical populations indicates that depression severity may be one of the correlates of suicidal behaviors across the depressive spectrum (40, 60, 61). Thus, while not retained in our final model, severity remains clinically important and should be considered when evaluating suicide risk in MDD patients.

Our study is also notable for its comprehensive inclusion of biological markers, particularly folate levels, which have not been extensively examined in many previous suicide risk models. Kim et al. (21) highlighted that low serum folate is associated with an increased risk of suicidality, aligning with our findings that suggest metabolic dysregulation may significantly impact suicide risk. Folate plays a critical role in the methylation of homocysteine into methionine, a process essential for neurotransmitter balance and overall mental health (14). Disruptions in this pathway due to folate deficiency can result in the accumulation of homocysteine, which is neurotoxic and may exacerbate depressive symptoms. Previous research has found elevated homocysteine to be associated with both depression and increased suicide risk (20). However, in our study, we did not observe a significant relationship between high homocysteine levels. This might be due to compensatory mechanisms in some patients that prevent homocysteine from rising, even when folate is low. These findings underscore the need for further research to clarify the role of folate and homocysteine in suicide risk.

Regarding treatment approaches, the follow-up data in our study revealed notable differences between patients with SA and those without. Patients with SA were significantly more likely to receive MECT compared to non-SA patients. This finding is consistent with previous research, which has shown that MECT is often used in severe cases of depression with suicidal behaviors (62, 63). Additionally, our findings indicated no difference in the use of antipsychotic medications between the two groups, aligning with the observation that both groups exhibited similar rates of psychotic symptoms. This supports the clinical perspective that antipsychotics primarily target psychotic symptoms rather than directly reducing suicide risk. Interestingly, mood stabilizer usage was significantly higher among SA patients. This aligns with findings from earlier studies, which noted that mood stabilizers can be particularly effective in managing the emotional dysregulation and impulsive behaviors commonly seen in individuals at higher risk of suicide (64, 65).

The strengths of this study include the use of a relatively large inpatient sample, integration of diverse data types (demographic, clinical, and biochemical), and the application of internal validation procedures to assess the robustness of the model. In addition, although our data were derived from a single Chinese inpatient sample, the consistency of several findings with studies from Western populations suggests that these correlates of suicidality may have cross-cultural relevance. However, several limitations should be acknowledged. First, due to the cross-sectional design, temporal and causal relationships between the included variables and SA cannot be established. Longitudinal studies are needed to confirm these associations and to better understand the temporal dynamics of suicide risk.

Second, standardized clinician- and self-rated depression scales (e.g., HAMD-17, MADRS) were not included because such scales were not yet fully digitized and therefore unavailable for extraction from medical records. Instead, depression severity was supplemented using ICD-10 diagnostic categories (moderate vs. severe). Given that the vast majority of our cohort consisted of inpatients with severe depression, the imbalanced distribution likely reduced statistical power, which may explain why severity was not retained in the final model. Accordingly, the applicability of our findings to patients with mild depression is limited. In addition, psychosocial context (e.g., social support, recent stressors), personality traits, and further biological markers such as inflammatory or metabolic parameters were not included, which may reduce the comprehensiveness of the analysis and omit important confounding or mediating factors. Third, although abnormal liver function was considered to control for its potential influence on biochemical markers, residual confounding from unmeasured variables remains possible. As electronic medical records become more comprehensive in the future, more nuanced and multidimensional data may become available, allowing for further optimization and refinement of the model. Fourth, the model was developed and evaluated in a single-center inpatient population, which may limit its generalizability to other clinical settings. Future multicenter, prospective studies are warranted to externally validate and refine this tool. Lastly, we excluded patients with suicidal ideation alone because documentation of ideation in the medical records varied greatly in detail and clarity, making reliable classification difficult in a retrospective design. This decision may have limited the generalizability of our findings to the broader spectrum of suicidal behaviors, as some patients with ideation may later SA.

## 5 Conclusion

In conclusion, this study identified several demographic, clinical, and biochemical variables—including tobacco and alcohol use, education level, stressful life events, and folate levels—that were significantly associated with SA in patients with MDD. By integrating these variables, we developed an nomogram that illustrates their relative contributions but should not be regarded as a validated clinical assessment tool. Future prospective and multicenter studies are warranted to confirm these associations and to evaluate the potential utility of such approaches in broader clinical settings.

## Data Availability

The raw data supporting the conclusions of this article will be made available by the authors, without undue reservation.
